# Amplatzer^™^ Vascular Plugs for Embolisation: A 10-Year Single-Centre Retrospective Study

**DOI:** 10.3390/jcm12216790

**Published:** 2023-10-27

**Authors:** Romaric Loffroy, Olivier Chevallier, Amin Mazit, Alexandre Malakhia, Marco Midulla

**Affiliations:** 1 Department of Vascular and Interventional Radiology, Image-Guided Therapy Center, François-Mitterrand University Hospital, 14 Rue Paul Gaffarel, BP 77908, 21079 Dijon, France; olivier.chevallier@chu-dijon.fr (O.C.); amin.mazit@chu-dijon.fr (A.M.); malakdebezak@hotmail.fr (A.M.); marco.midulla@chu-dijon.fr (M.M.); 2 ICMUB Laboratory, UMR CNRS 6302, 9 Avenue Alain Savary, 21000 Dijon, France

**Keywords:** Amplatzer^™^ Vascular Plug, AVP, plug, embolisation, interventional radiology

## Abstract

Our objective was to investigate the indications, effectiveness, and safety of Amplatzer^™^ Vascular Plugs (AVPs) in clinical practice. To retrospectively identify patients managed with AVPs at the Dijon University Hospital between January 2011 and April 2021, we searched materials vigilance registries and procedure reports. The 110 identified patients underwent 111 procedures with delivery of 202 AVPs into 118 vessels; 84% of the procedures were performed by radiologists with over 10 years’ experience and 67% were scheduled. Varicocele, haemostasis, pelvic varicose veins, and arterio-venous dialysis fistulas accounted for 69% of procedures. The technical and clinical success rates were 99% and 97%, respectively. The single major complication was AVP migration in a high-flow internal iliac vein, with no residual abnormalities after successful device retrieval. Several AVPs and/or concomitant injection of coils or liquid agents were used in 80% of cases. The use of AVPs alone occurred chiefly for splenic artery embolisation in trauma patients and for collateral vein occlusion in dysfunctional arterio-venous dialysis fistulas. No cases of recanalisation occurred during the 19 ± 29 month follow-ups. Based on their good safety and effectiveness profile, AVPs deserve to be part of the therapeutic armamentarium of every interventional radiologist.

## 1. Introduction

Since embolisation was first described in 1972 [1], interventional radiology has developed steadily, earning a major role in the treatment of many conditions [2]. Embolisation aims to occlude one or more vessels by inducing formation of a clot [3]. Available embolic materials include mechanical devices, particles, foams, and liquids.

The Amplatzer™ Vascular Plugs (AVPs; Abbott Cardiovascular, Chicago, IL, USA) are among the non-absorbable mechanical embolisation devices, together with coils and occlusion balloons. They were derived from the septal occlusion plugs initially developed for congenital heart defects by Dr Kurt Anton Amplatz [4,5]. There are now four AVP models, each with its unique size, conformation, delivery method, and thrombogenicity (Table 1) [6]. All models are made of nitinol braid. AVPs can be placed accurately and, compared to coils, have a low risk of migration, notably in high-flow vessels [3,6], due to the greater radial force exerted by the device on the vessel wall. The manufacturer recommends that AVPs should be larger than the target vessel by approximately 30% for arteries and 50% for veins. When the AVP is in the correct position, it is released by rotating the cable counterclockwise. Operators must be conversant with the advantages and disadvantages of each model and of its place within embolisation strategies. AVPs have no contraindications and appear suitable for a variety of situations [7].

There are a few studies available collecting data of actual clinical uses of AVPs in a large panel of indications [4,6,7]. Therefore, the aim of this retrospective single-centre observational study was to assess the use, indications, clinical effectiveness, and safety of AVPs implanted over a recent 10-year period at a high-volume centre.

## 2. Materials and Methods

### 2.1. Patient Selection

Eligible patients were those managed by AVP implantation between January 2011 and April 2021 at the Dijon University Hospital, Dijon, France. Patients were identified retrospectively via materials vigilance registries and by searching procedure reports in the hospital electronic patient records for the keywords “plug”, “plugs”, “AVP”, “AVPs”, and “embolisation”. All study data were collected by a single investigator. All patients who underwent embolisation with at least one AVP were included. There were no exclusion criteria.

For each patient, the following were recorded: age and sex; date of the procedure; indication; arterial or venous approach; target vessel; delivery via a catheter vs. an introducer sheath; combination with other embolisation materials, with their type; AVP size and model; technical and clinical success rates; operator; coagulation disorders; complications; follow-up duration; and whether embolisation was emergent, high-priority, or scheduled.

### 2.2. AVP Delivery Technique

Most of the embolisation procedures were performed under local anaesthesia, by several operators, through different arterial or venous routes. The AVP delivery methods were selected by the operators. Most of the AVPs were delivered through a 6 or 7 Fr sheath or 5 Fr catheters. If needed, additional mechanical or liquid embolic agents were used via a standard coaxial technique.

### 2.3. Outcomes and Follow-Up

We defined technical success as proper AVP position in the target vessel with angiography-documented occlusion or substantial flow reduction as assessed by the operator. Clinical success was defined as resolution of the symptoms that prompted embolisation, as reported by the operator, referring physician, or patient. Complications were classified as major when they increased hospital-stay length or required a new procedure. Finally, the embolisation procedure was considered emergent if it was performed as soon as possible due to immediately life-threatening symptoms, high-priority if performed promptly due to major but not immediately life-threatening symptoms, and scheduled in other situations. Follow-up time was determined for each patient from the time from embolisation to last visit and/or phone call available in the range of the study period. Control imaging, either US or CT, was available in all patients at mean follow-up to confirm results. Long-term clinical results were assessed by a multidisciplinary team including mainly IRs and clinical specialists.

### 2.4. Statistical Analysis

Quantitative variables are described as mean ± SD or median [interquartile range] and qualitative variables as numbers (%). The number of procedural failures was too small to allow a statistical analysis of risk factors for procedural failure.

## 3. Results

### 3.1. Patients

**Figure 1:**
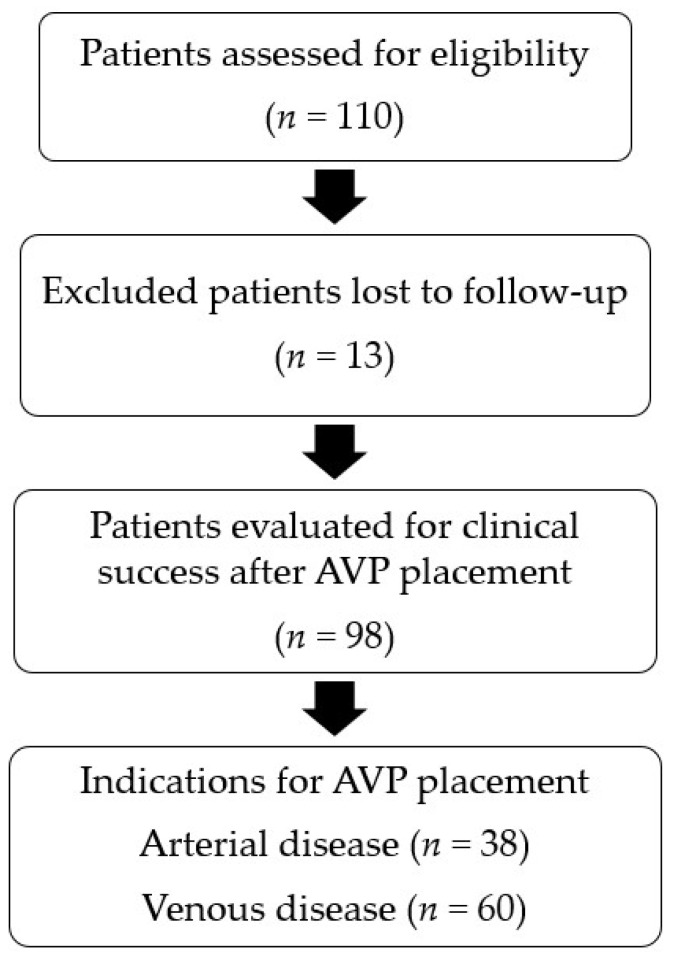
Flowchart of the study.

Over the 10-year period, 202 AVPs were delivered within 118 vessels during 111 embolisation procedures in 110 patients, as shown by the flowchart (Figure 1). Three radiologists with more than 10 years’ experience as a senior doctor performed 93 (84%) of the 111 procedures. The remaining 18 procedures were performed by 13 radiologists with less than 5 years’ experience as a senior. The data needed to evaluate technical success were available for all 111 procedures. In total, 13 (11.8%) of the 110 patients, each with a single procedure for varicocele, were lost to follow-up, leaving 98 procedures for the evaluation of clinical success. Mean follow-up was 19 ± 29 months and median follow-up was 4 months (IQR, 1–34 months).

Table 2 reports the main patient features at baseline. Of the five patients with coagulopathy, one was taking anticoagulant therapy and required emergent embolisation for life-threatening bleeding and the other four had liver failure and required high-priority embolisation.

### 3.2. Indications and Techniques

At our centre, the AVP models and sizes listed in Table 3 were available. The AVP model, size, and delivery method were selected by the operator during the procedure based on angiography findings. The 8 mm AVP 4 was the most often used (61/202, 30.2%).

Table 4 lists the numbers of procedures for each target vessel. The most common targets were the gonadal veins, followed by the splenic arteries, then by the internal iliac veins.

**Figure 2:**
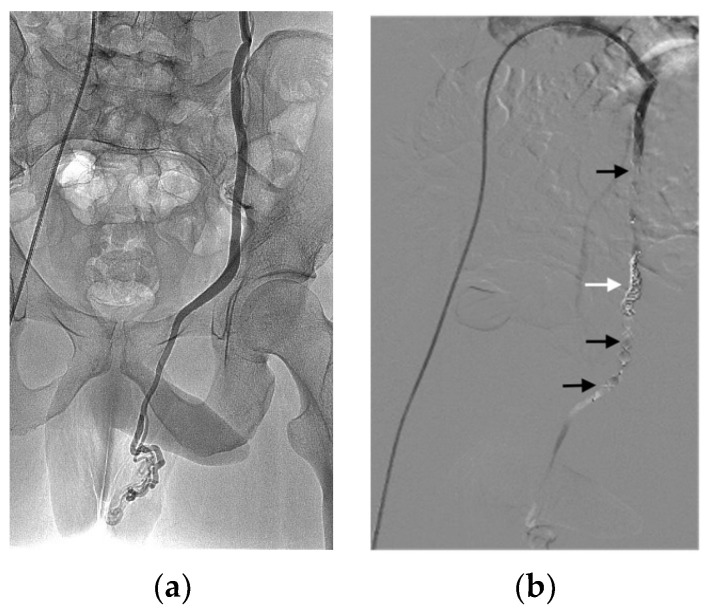
Embolisation of a painful left varicocele in a 28-year-old patient using achieved by combining an 8 mm AVP IV, a 10 mm AVP II, two 8 mm coils (white arrow), and a 16 mm AVP II. AVPs are indicated by black arrows. (**a**) Angiogram shows left varicocele. (**b**) Control angiogram showing the occlusion of the varicocele.

Table 5 reports the main characteristics and indications of the 111 procedures. Only six procedures were performed under general anaesthesia. All vascular approaches were performed using the Seldinger technique under ultrasound guidance. We identified 12 different indications (Table 5), of which the most common was varicocele causing pain and/or impaired fertility illustrated in Figure 2 [8,9,10,11].

**Figure 3:**
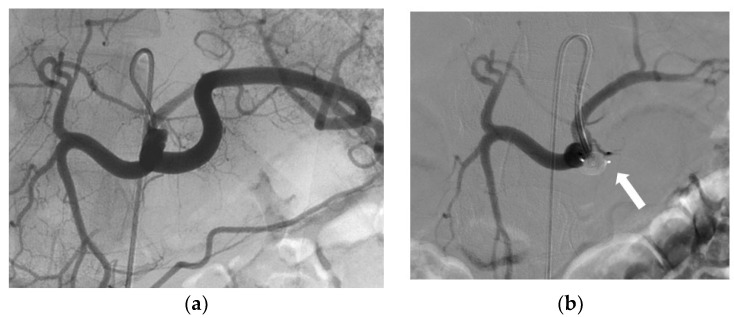
Preventive proximal splenic artery embolisation using in an 8 mm AVP 4 (white arrow) in an 18-year-old traffic-accident victim. (**a**) Angiogram of coeliac trunk. (**b**) Control angiogram showing the occlusion of the splenic artery.

An AVP was routinely positioned in the upper third of the gonadal vein. When the anatomical configuration was favourable, a second AVP was placed opposite the iliopectineal line, whereas in patients with vascular tortuosity or early small branching, coils were used. Of the 103 AVPs placed in gonadal veins, 26 were 8 mm AVP 4; the 5 mm, 6 mm, and 7 mm AVP 4 devices were used 4, 5, and 7 times, respectively; and the 10 mm, 12 mm, 14 mm, and 16 mm AVP II devices were used 16, 15, 8, and 12 times, respectively. The second most common indication was bleeding and haemostasis control (Table 5). For this indication, the most common target vessel was the proximal splenic artery (13/19), which was embolised to reduce flow while preserving splenic vascularisation via the pancreatic arches (Figure 3) [12,13,14,15,16,17,18,19]. All spleen injuries were grade IV in the American Association for the Surgery of Trauma classification. The other target arteries for bleeding or haemostasis control were the carotid (*n* = 2), renal (*n* = 2), gastroduodenal (*n* = 1), and internal pudendal (*n* = 1) arteries. Active bleeding, if present, was managed by additional hyper-selective embolisation. The most commonly used AVP for haemostasis was the 8 mm AVP 4 (13/23, 57%), in keeping with the average size of splenic arteries [20]; the 7 mm AVP 4 was used four times, the 10 mm AVP II three times, and the 12 mm AVP II three times.

The AVP delivery methods were selected by the operators. The most common were a 6 Fr introducer (73/202), 5 Fr Cobra catheter (31/202), 7 Fr introducer (25/202), and 5 Fr spinal catheter (23/202). For 10 of the 202 AVPs, data on the method of delivery were unavailable.

AVPs were used in combination with another type of embolic material in 94 (80%) vessels and alone in 24 (20%) vessels. Table 6 shows the combinations. The most common materials used in addition to the AVP were a second AVP and/or coils. The use of combinations was decided by the operator and intended to increase the efficiency and speed of occlusion.

### 3.3. Effectiveness

The technical success rate was 99% (110/111 procedures). The only technical failure consisted in AVP migration in a young patient undergoing embolisation of a large arterio-venous malformation. The clinical success rate was 97% (95/98 procedures). The three clinical failures were persistent vascularisation of a large pelvic arterio-venous malformation, persistently patent collateral requiring a second procedure in a patient treated for pelvic varicose veins, and patent collateral with an independent ostium in a patient with varicocele and vascular malformations. No cases of recanalisation after embolisation were recorded during follow-up.

### 3.4. Safety

Six major complications (6.1%) occurred during the study period. Of these, five do not seem directly related to the use of AVPs, including false femoral aneurysms at the arterial puncture site in three patients and one case each of extensive thrombosis after embolisation of a porto-renal varicose vein and of cardiorespiratory failure in a patient with a splenic injury. The remaining major complication was directly related to the use of an AVP and resulted in the only technical failure: the device migrated during embolisation of a large pelvic arterio-venous malformation with high cardiac output. The 22 mm AVP II was placed within the internal iliac vein to induce a flow reduction enabling nidus embolisation with a liquid agent. Device size was in keeping with manufacturer recommendations. However, the high venous blood flow caused gradual device migration during the procedure. The operator had time to insert an inferior-vena-cava filter to prevent migration into the pulmonary artery. The AVP was then recovered using a lasso and the filter was removed afterwards in the same session. The patient underwent a successful repeat procedure with placement of an arterial occlusion balloon to reduce flow and allow embolisation with liquid agents.

Two minor complications (1.8%) were recorded, a puncture-site haematoma and patient-reported discomfort from AVPs used to treat varicocele. There were both resolved within a few weeks without treatment. All complications are listed in Table 7.

## 4. Discussion

Our experience with 202 AVPs support the safety and effectiveness of these devices. The technical and clinical success rates were 99% and 97%, respectively. No cases of recanalisation were recorded during the mean follow-up of 19 ± 29 months. The rare failures were related to incomplete initial embolisation of non-visualised and consequently untreated collaterals responsible for recurrent symptoms. The only major complication was AVP migration due to high flow, with successful retrieval of the device.

The absence of recanalisation in this study is consistent with the previous reporting of only rare cases, predominantly in high-flow vessels [21,22,23]. Methods to avoid recanalisation include the coil-in-plug technique [24,25,26] and combined use of coils [27]. The combination of other embolic methods to one or more AVPs in our population may explain why no patient experienced recanalisation.

A retrospective review of 23 consecutive patients of vascular embolisation using AVPs in a variety of different clinical settings was reported by Tuite et al. [5]. The AVPs were chosen to have a diameter approximately 30–50% greater than the target vessel. Additional embolic agents were used in some cases. All target vessels were successfully occluded with no device malpositioning or malfunction. In 14 (61%) patients the AVP was the sole embolic material. In the remaining patients, additional agents were used, particularly in preoperative embolisation of highly vascular renal tumours. In another study, 14 patients had proximal splenic artery embolisation performed with AVPs [16]. Device placement in the desired location was successful in all cases, with device repositioning required in two. Additional coils placed in three patients could all be packed into a tight configuration. A second AVP was placed in one patient. There were no complications of the procedures. Follow-up CT images showed no evidence of migration or recanalisation of any of the devices.

Combining AVPs with other embolic materials is also useful for decreasing the time to vessel occlusion, notably in emergencies. When prompt occlusion is required, procedure duration and radiation exposure are the key variables. Vessel occlusion after AVP insertion requires several minutes, during which the vessel remains patent by angiography. Waiting until occlusion occurs before performing the angiographic control increases procedure duration and radiation dose to the patient, especially if multiple controls are required. Adding another type of embolic material to achieve faster embolisation was the strategy used for most procedures in our study. One technical failure was ascribed to high flow rate within the vessel in a patient treated for AVM. Some adjustments can be made in these specific cases to prevent these outcomes by adding other embolic agents, especially liquids such as cyanoacrylates if possible and safe from a technical point of view. A comparison of occlusion times with AVPs and other embolic materials used in clinical practice would be of interest. 

AVP migration is a rare complication. In the only case in our study, an AVP was inserted into the internal iliac vein to treat a pelvic arterio-venous malformation. AVP size was selected according to manufacturer recommendations. The migration can be ascribed to the very high blood flow rate within the vein. Operators should exercise particular caution when using AVPs to embolise high-flow vessels. None of the arterial procedures in our study were followed by complications. However, several cases of AVP migration within arteries have been reported [28,29].

AVPs have been proven useful in several indications. In patients with splenic injuries and favourable anatomical features, AVP embolisation alone has been reported to effectively occlude the splenic artery with a lower risk of migration compared to coils [12,14]. However, the indications for splenic artery embolisation in splenic injuries are highly controversial, and practices regarding the use of this procedure may consequently vary across centres. In dysfunctional arterio-venous dialysis fistulas with flow theft by a venous collateral, AVPs have been found simple and effective for embolising the collateral to restore sufficient flow in the main drainage vein [30,31,32]. However, long-term follow-up data for this indication are needed. For the treatment of varicocele, AVPs are used only in combination with other embolisation materials, which increases the cost and duration of the procedure. Moreover, AVPs occlude only the main vein and not accessory draining veins, which can lead to varicocele recurrence, as seen in one of our patients. At our centre, we now use cyanoacrylate to treat varicocele in order to increase the likelihood of occluding not only the main vein but also any possible accessory drainage veins. Finally, AVPs can be used to anchor other embolisation materials, notably coils, which have a higher risk of migration [33]. This method is particularly useful for the treatment of high-flow vessels. The AVP is inserted first to decrease the flow rate, and placement of another AVP or coils then achieves complete occlusion.

A major limitation of our study is the retrospective design. More specifically, technical failures may not have been routinely recorded in the patient files, and our technical success rate may therefore have been overestimated. Also, time to vessel occlusion after AVP release was not recorded. Indeed, technical success of embolisation with AVPs is not always easy to report since it can be used in combination with other embolic agents. In that case, technical success can be also ascribed to the combination. In our experience so far, the used of additional embolic agents is probably not necessary in most cases if we wait enough time after AVP embolisation for final control.

The indications for AVP embolisation varied widely. Moreover, some of the recognised indications for AVPs were not present in any of our patients, such as the pulmonary arterio-venous malformations [34,35] and endovascular aortic aneurysm repair with internal iliac artery occlusion [36]. The technical and clinical success rates reported here cannot be extrapolated to these indications. However, this study allows us to underline the range of potential indications where AVPs can be used as an embolic agent, alone or in combination with other embolic agents, emphasising the versatility of this specific agent in several clinical scenarios, and adding new data to the existing literature on this topic. Another limitation was the absence of data on the delivery method for 10 patients. Last, 13 (11.8%) of the 110 patients were lost to follow-up.

In our single-centre study, the vast majority of the procedures were performed by highly experienced operators, and our results may not apply to centres with smaller caseloads. Finally, we did not collect data on the AVP models I and III because these models were not available at our institution.

## 5. Conclusions

The data reported here strongly support the safety and effectiveness of AVPs across a spectrum of indications in everyday clinical practice. They confirm that rapid implantation and a low risk of migration are major advantages of AVPs. Furthermore, no case of recanalisation was reported during the follow-up period. Combining AVPs with other embolic materials proved successful in various indications. An important caveat is that most procedures were performed by experienced operators at our high caseload centre. Selection of the optimal AVP size and accurate positioning of the device can be challenging, notably in the event of marked vessel tortuosity. Continued reviews of AVP embolisation in clinical practice are needed to further refine the indications and techniques.

## Figures and Tables

**Table 1:** Main features of the four models of Amplatzer Vascular Plug (AVP) [6].

	AVP I	AVP II	AVP III	AVP 4
Configuration	One cylindrical module	Three cylindrical modules	Oblong with two enlarged ends	Two profiled modules
Advantages and disadvantages	Short anchoring area, high radial force Low thrombogenic capacity at high flow rates	Rapid occlusion, lower risk of migration and recanalisation Long anchoring area	Faster occlusion, especially in high-flow vessels.	Can be used in tortuous anatomy and in medium calibre vessels. Delivered with diagnostic probes
Diameter (mm)	4–16	3–22	4–14 (long axis)	4–8
Introducer sheath	4 Fr: 4/6/8 mm 5 Fr: 10/12 mm 6 Fr: 14/16 mm	4 Fr: 3/4/6/8 mm 5 Fr: 10/12 mm 6 Fr: 14/16 mm 7 Fr: 18/20/22 mm	4 Fr: 4/6 mm 5 Fr: 8/10 mm 7 Fr: 12/14 mm	Diagnostic probe 0.038
Guiding catheter	5 Fr: 4/6/8 mm 6 Fr: 10/12 mm 8 Fr: 14/16 mm	5 Fr: 3/4/6/8 mm 6 Fr: 10/12 mm 8 Fr: 14/16 mm 9 Fr: 18/20/22 mm	6 Fr: 4/6 mm 7 Fr: 8/10 mm 9 Fr: 12/14 mm	4 or 5 Fr depending on the probe model
Unconstrained length (mm)	7–8	6–18	6.5	10–13.5
Maximum length to be delivered (cm)	100	100	120	100

**Table 2:** Characteristics of the 110 study patients.

Features	Median (Range) or No. (%)
Age, years	43 (30–66)
Males/Females	88 (80%)/22 (20%)
Coagulation disorders	5 (6.8%)
aPTT > 1.5	2 (1.8%)
INR > 3	2 (1.8%)
aPTT > 1.5 and INR > 3	1 (0.9%)

aPTT: activated partial thromboplastin time; INR: international normalised ratio.

**Table 3:** Model and size of the 202 Amplatzer Vascular Plugs delivered during the 111 procedures.

Model and Size	Number of AVPs (%)
AVP IV	101 (50)
5 mm	7 (3.5)
6 mm	10 (4.9)
7 mm	23 (11.4)
8 mm	61 (30.2)
AVP II	101 (50)
10 mm	31 (15.3)
12 mm	24 (11.9)
14 mm	12 (5.9)
16 mm	15 (7.4)
18 mm	8 (4.0)
20 mm	4 (2.0)
22 mm	7 (3.5)

**Table 4:** The 118 target vessels.

Arteries	*n* = 39 (33%)	Veins	*n* = 79 (67%)
Splenic	18 (15.3)	Gonadal	44 (37.3)
Renal	4 (3.4)	Internal iliac	8 (6.8)
Gastro-duodenal	3 (2.5)	TIPS ^1^	7 (5.9)
Gluteal	3 (2.5)	Cephalic	5 (4.2)
Carotid	2 (1.7)	Porto-hepatic fistula	2 (1.7)
Deep femoral	2 (1.7)	Basilic	2 (1.7)
Main hepatic	2 (1.7)	Humeral	2 (1.7)
Other	5 (4.2)	Other	9 (7.6)

^1^ TIPS: trans-jugular intrahepatic porto-systemic shunt.

**Table 5:** Characteristics and indications of the 111 procedures.

Approach	N (%)
Arterial	38 (34)
Venous	73 (66)
Degree of urgency	
Emergent ^a^	19 (17)
High-priority ^b^	18 (16)
Scheduled ^c^	74 (67)
Indications	
Varicocele	33 (29.8)
Haemostasis	19 (17.1)
Pelvic varicose veins	12 (10.8)
Collaterals of arteriovenous dialysis fistulas	12 (10.8)
Arterial aneurysm	8 (7.2)
Occlusion/recalibration TIPS ^1^	7(6.3)
Arterio-venous fistula	6 (5.4)
Hepatic arterial infusion	5 (4.5)
Arterio-venous malformation	4 (3.6)
Varicose veins due to portal hypertension	3 (2.7)
Other	2 (1.8)

^1^ TIPS: trans-jugular intrahepatic porto-systemic shunt; ^a^ mainly bleeding patients; ^b^ mainly portal hypertension-related complications; ^c^ all other patients.

**Table 6:** Embolic materials used in the 118 vessels.

Utilisation	N (%)
One AVP	24 (20%)
One AVP combined with	94 (80%)
Coils	19
1 other AVPs + coils	16
1 other AVP	13
1 other AVP + Polidocanol	11
Polidocanol	6
Cyanoacrylate	6
1 other AVP + Polidocanol + coils	5
Polidocanol + coils	5
Coils + cyanoacrylate	4
Stent	4
1 other AVP + cyanoacrylate	3
1 other AVP + stent	1
Particles	1
N of materials used in combination with AVPs (*n* = 94)	
AVP	49
Coils ^a^	49
Polidocanol ^b^	27
Cyanoacrylate ^c^	13
Stent	5
Particles ^d^	1

^a^ Concerto^TM^ Coils, Medtronic, CA, USA; ^b^ Aethoxysklerol^®^, KreusslerPharma, Paris, France; ^c^ Glubran^®^2, GEM, Viareggio, Italy; ^d^ Embosphere^®^ Microspheres, MeritMedical, UT, USA.

**Table 7:** Postoperative complications in the 98 patients evaluated.

Complications	N (%)
Total	8 (8.2)
Minor	2 (2)
Puncture-site hematoma	1 (1)
Patient-reported discomfort	1 (1)
Major	6 (2.5)
Unrelated to AVP use	5 (5.1)
False aneurysm at the puncture site	3 (3.1)
Extensive porto-renal varicose vein thrombosis	1 (1)
Cardiorespiratory failure	1 (1)
Related to AVP use	1 (1)
AVP migration into the IVC requiring removal	1 (1)

IVC, inferior vena cava.

## Data Availability

The data presented in this study are available on request from the corresponding author. The data are not publicly available due to identity reasons.

## References

[1] Rösch J., Dotter C.T., Brown M.J. (1972). Selective arterial embolization: A new method for control of acute gastrointestinal bleeding. Radiology.

[2] Rousseau H., Vernhet-Kovacsik H., Mouroz P.R., Otal P., Meyrignac O., Mokrane F.Z. (2019). Future of interventional radiology. Presse Med..

[3] Medsinge A., Zajko A., Orons P., Amesur N., Santos E. (2014). A case-based approach to common embolization agents used in vascular interventional radiology. Am. J. Roentgenol..

[4] Sharafuddin M.J.A., Gu X., Urness M., Amplatz K. (1999). The nitinol vascular occlusion plug: Preliminary experimental evaluation in peripheral veins. J. Vasc. Interv. Radiol..

[5] Tuite D.J., Kessel D.O., Nicholson A.A., Patel J.V., McPherson S.J., Shaw D.R. (2007). Initial clinical experience using the Amplatzer Vascular Plug. Cardiovasc. Interv. Radiol..

[6] Wang W., Li H., Tam M.D., Zhou D., Wang D.X., Spain J. (2012). The Amplatzer Vascular Plug: A review of the device and its clinical applications. Cardiovasc. Interv. Radiol..

[7] Güneyli S., Çinar C., Bozkaya H., Parıldar M., Oran İ. (2014). Applications of the Amplatzer Vascular Plug to various vascular lesions. Diagn. Interv. Radiol. Ank. Turk..

[8] Johnson D., Sandlow J. (2017). Treatment of varicoceles: Techniques and outcomes. Fertil. Steril..

[9] Masson P., Brannigan R.E. (2014). The varicocele. Urol. Clin. N. Am..

[10] Halpern J., Mittal S., Pereira K., Bhatia S., Ramasamy R. (2016). Percutaneous embolization of varicocele: Technique, indications, relative contraindications, and complications. Asian J. Androl..

[11] Sheehan M., Briody H., O’Neill D.C., Bowden D., Davis N.F., Given M., Mohan P., Lee M.J. (2020). Pain relief after varicocele embolization: The patient’s perspective. J. Med. Imaging Radiat. Oncol..

[12] Zhu X., Tam M.D.B.S., Pierce G., McLennan G., Sands M.J., Lieber M.S., Wang W. (2011). Utility of the Amplatzer Vascular Plug in splenic artery embolization: A comparison study with conventional coil technique. Cardiovasc. Interv. Radiol..

[13] Ahuja C., Farsad K., Chadha M. (2015). An overview of splenic embolization. Am. J. Roentgenol..

[14] Jambon E., Hocquelet A., Petitpierre F., Le Bras Y., Marcelin C., Dubuisson V., Grenier N., Cornelis F. (2018). Proximal embolization of splenic artery in acute trauma: Comparison between Penumbra occlusion device versus coils or Amplatzer Vascular Plug. Diagn. Interv. Imaging.

[15] Wang W., Tam M.D., Spain J., Quintini C. (2013). Gelfoam-assisted Amplatzer Vascular Plug technique for rapid occlusion in proximal splenic artery embolization. AJR Am. J. Roentgenol..

[16] Widlus D.M., Moeslein F.M., Richard H.M. (2008). Evaluation of the Amplatzer Vascular Plug for proximal splenic artery embolization. J. Vasc. Interv. Radiol. JVIR.

[17] Coccolini F., Montori G., Catena F., Kluger Y., Biffl W., Moore E.E., Reva V., Bing C., Bala M., Fugazzola P. (2017). Splenic trauma: WSES classification and guidelines for adult and pediatric patients. World J. Emerg. Surg. WJES.

[18] Ng E.H., Comin J., David E., Pugash R., Annamalai G. (2012). Amplatzer Vascular Plug 4 for proximal splenic artery embolization in blunt trauma. J. Vasc. Interv. Radiol. JVIR.

[19] Gheju I., Venter M.D., Beuran M., Gulie L., Racoveanu I., Carstea P., Iftimie Nastase I., Venter D.P. (2013). Grade IV blunt splenic injury--the role of proximal angioembolization. A case report and review of literature. J. Med. Life.

[20] Brinkman D.J., Troquay S., de Jonge W.J., Irwin E.D., Vervoordeldonk M.J., Luyer M.D.P., Nederend J. (2021). Morphometric analysis of the splenic artery using contrast-enhanced computed tomography (CT). Surg. Radiol. Anat..

[21] Dorenberg E.J., Hafsahl G., Andersen R., Krohg-Sørensen K. (2006). Recurrent rupture of a hypogastric aneurysm caused by spontaneous recanalization of an Amplatzer Vascular Plug. J. Vasc. Interv. Radiol..

[22] Gómez-Martínez P., Ciampi Dopazo J.J., González Fejás A., Lanciego C. (2014). Recanalización espontánea tras la embolización de una arteria renal con un tapón vascular Amplatzer tipo 4. Radiología.

[23] Fidelman N., Gordon R.L., Bloom A.I., LaBerge J.M., Kerlan R.K. (2008). Reperfusion of pulmonary arteriovenous malformations after successful embolotherapy with vascular plugs. J. Vasc. Interv. Radiol. JVIR.

[24] Koganemaru M., Tanoue S., Kuhara A., Kugiyama T., Abe T. (2019). Internal coil packing method for the Amplatzer vascular plug 4. Diagn. Interv. Radiol..

[25] Nagatomi S., Ichihashi S., Yamamoto H., Bolstad F., Kichikawa K. (2021). Coil-in-plug technique using the Amplatzer Vascular Plug II to occlude a portosystemic shunt. Vasc. Endovasc. Surg..

[26] Chegai F., Gandini R. (2019). Intraplug coils delivery for fast closure of giant arteriovenous fistulas (AVFs) aneurysm in dialyzed patient. Radiol. Case Rep..

[27] Trerotola S.O., Pyeritz R.E. (2010). Does use of coils in addition to Amplatzer Vascular Plugs prevent recanalization?. AJR Am. J. Roentgenol..

[28] Maldonado Fernández N., López Espada C., Linares Palomino J.P., Pérez Vallecillos P., García Róspide V. (2020). Migration and surgical retrieval of an Amplatzer Septal Occluder into abdominal aorta. Ann. Vasc. Surg..

[29] Maleux G., Rega F., Heye S., Troost E., Budts W. (2011). Asymptomatic migration of a first-generation Amplatzer Vascular Plug into the abdominal aorta: Conservative management may be an option. J. Vasc. Interv. Radiol..

[30] Ozyer U., Aytekin C., Yildirim U.M., Harman A., Karakayali F., Boyvat F. (2011). Use of the Amplatzer^®^ Vascular Plug II in endovascular occlusion of dialysis shunts with tributary veins. J. Vasc. Access.

[31] Ahmed O., Patel M., Ginsburg M., Jilani D., Funaki B. (2014). Effectiveness of collateral vein embolization for salvage of immature native arteriovenous fistulas. J. Vasc. Interv. Radiol. JVIR.

[32] Zangan S.M., Falk A. (2009). Optimizing arteriovenous fistula maturation. Semin. Interv. Radiol..

[33] Hoit D.A., Schirmer C.M., Malek A.M. (2006). Use of the Amplatzer Vascular Plug as an anchoring scaffold for coil-mediated parent vessel occlusion: Technical case report. Neurosurgery.

[34] Lee S.Y., Lee J., Kim Y.H., Kang U.R., Cha J.G., Lee J., Cha S.I., Kim C.H. (2019). Efficacy and safety of Amplatzer Vascular Plug Type IV for embolization of pulmonary arteriovenous malformations. J. Vasc. Interv. Radiol. JVIR.

[35] Tapping C.R., Ettles D.F., Robinson G.J. (2011). Long-term follow-up of treatment of pulmonary arteriovenous malformations with Amplatzer Vascular Plug and Amplatzer Vascular Plug II devices. J. Vasc. Interv. Radiol. JVIR.

[36] Vandy F., Criado E., Upchurch G.R., Williams D.M., Rectenwald J., Eliason J. (2008). Transluminal hypogastric artery occlusion with an Amplatzer Vascular Plug during endovascular aortic aneurysm repair. J. Vasc. Surg..

